# Editorial: The Human Microbiome and Cancer

**DOI:** 10.3389/fmicb.2020.01514

**Published:** 2020-07-21

**Authors:** Gary P. Moran, Nezar Al-Hebshi

**Affiliations:** ^1^School of Dental Science, Dublin Dental University Hospital and Trinity College Dublin, Dublin, Ireland; ^2^Kornberg School of Dentistry, Temple University, Philadelphia, PA, United States

**Keywords:** cancer, microbiome, bacteria, virus, fungus

The role of microorganisms in carcinogenesis has been a hotly contested area of research for over a century. Early pioneers such as Peyton Rous were the first to demonstrate that viral pathogens could initiate transformation of avian cells (Rous, 1910). Since then, the roles of viral pathogens such as Epstein-Barr virus and human papilloma virus in carcinogenesis have been well-established (Sarid and Gao, 2011). More recently, the role of the bacterium *Helicobacter pylori* in the etiology of gastric cancer has been elucidated (Alfarouk et al., 2019) and compelling evidence for a fungal etiology in pancreatic cancer has been proposed (Aykut et al., 2019). A role of the endogenous microbiota in the etiology and progression of human cancers is now under intense investigation (Al-Hebshi et al., 2019; Elinav et al., 2019; Healy and Moran, 2019). Advances in DNA sequencing have opened up a new frontier in microbiome analysis leading to an explosion in studies to analyse changes in human bacterial, fungal, and viral communities during the development of human cancers. Metabolomics also allows us to study the impact of microbial metabolites (e.g., acetaldehyde) on human physiology (Amer et al., 2020). These studies aim not only to identify novel etiologic agents, but to determine if microbiome changes could have diagnostic potential or whether the microbiome has a direct role in malignant progression, metastatic spread, or the success of chemotherapeutic regimens. Most studies focusing on the human microbiome and cancer have investigated the role of the bacteriome on mucosal surfaces in the gastrointestinal tract. The role of the microbiome in non-mucosal cancers such as breast and prostate cancers is also under investigation (Sfanos et al., 2017; Chen et al., 2019). This Research Topic brings together a collection of studies investigating the microbiome of the oral and gastrointestinal (GIT) microbiomes, combining original research and up to date reviews.

One of the most important aspects of a microbiome study is DNA extraction and in the first chapter of this collection, Zhang C. et al. explore whether homogenization or enzymatic lysis of microbiome samples affects microbiome profiles in endoscopic biopsy samples. The authors conclude that although both methods produce similar profiles, homogenization provides higher microbial DNA content.

One area where microbiome profiling could potentially improve patient outcomes is as a diagnostic tool to identify those at high risk of malignant transformation or to identify the stage of cancer development. The diagnostic potential of the gut microbiome is highlighted by Ni et al. who develop a measurement of microbial disturbance, termed dysbiosis index (*D*_*dys*_), based on changes to the fecal microbiota in hepatocellular carcinoma patients. This dysbiosis index may have the potential to diagnose those with the disease and may in the future determine prognosis at different stages of hepatocellular carcinoma (Ni et al.).

The collection continues with several studies of the oral microbiome. The oral cavity is one of the most diverse areas of the human GIT and an increasing number of studies are demonstrating the increased abundance of *Fusobacterium* spp. in the development of OSCC (Al-hebshi et al., 2017; Amer et al., 2017). Zhang Z. et al. show that enrichments for *Fusobacterium* spp. are specific to tumor sites in OSCC patients, with reduced levels observed in saliva and oral rinse specimens from the same patients. Kageyama et al. extend the analysis of the salivary microbiome to include patients with diverse cancers throughout the GIT. This study also demonstrates elevated levels of *F. nucleatum* in patients with oropharyngeal cancers and identifies a general enrichment of *P. gingivalis* in all patients with cancers of the digestive tract (Kageyama et al.). The influence of the oral microbiome may also extend beyond the oral cavity, as shown by Chen X-H. et al., who demonstrate that gastric cancers exhibit increased abundance of taxa normally associated with the oral microbiome. The presence of *H. pylori* in these gastric samples also impacts upon the composition of these communities, suggesting that *H. pylori* may have a central role in the development of dysbiosis. The involvement of *H. pylori* the development of gastric biofilms is further explored by Rizatto et al., who discuss the potential role and mechanisms of biofilm formation in the gastric mucosa and identify future directions for this research area.

The intestinal microbiota is under intense investigation for its roles in regulating metabolism, immunity, and the interaction with cancer cells. In addition, specific pathogens have been implicated in carcinogenesis including *F. nucleatum* and *Streptococcus gallolyticus* subsp. *Gallolyticus*. Ma et al. present a timely and comprehensive review of the impact of the gut microbiome on cancer chemotherapy. Continuing this topic, Cong, Zhu, Zhang et al. present novel data showing the impact of chemotherapy on microbial networks in the intestinal microbiota, opening up the possibility to explore how these changes impact upon chemotherapeutic outcomes. The same authors also investigate the effect of surgical interventions on the gut microbiome of patients with CRC (Cong, Zhu, Liu et al.). These data show that surgical interventions have a strong impact on the microbiome resulting in increased levels of *Klebsiella* spp., which was significantly linked with lymphatic invasion.

Targeted interventions to restore healthy or beneficial microbiomes are still in their infancy. Here, Wu et al. present exciting data showing that chitooligosaccharides protect mice from colorectal carcinomas by reducing the levels of *Escherichia-Shigella, Enterococcus*, and *Turicibacter* and by promoting the growth of butyrate producing bacteria.

In summary, this collection offers an insight into the developing area of microbiome research in oncology, and highlights the growing potential of these scientific tools for improving diagnosis and treatment of these devastating diseases.

## Author Contributions

The manuscript was written and prepared by GM and NA-H. All authors contributed to the article and approved the submitted version.

## Conflict of Interest

The authors declare that the research was conducted in the absence of any commercial or financial relationships that could be construed as a potential conflict of interest.
